# Effectiveness of Er:YAG Laser Combined with Photobiomodulation on Periodontitis Based on 3-month Observation

**DOI:** 10.3290/j.ohpd.b2960543

**Published:** 2022-04-27

**Authors:** Bin Yang, Huifen Li, Peiqing Zhang, Binpin Wang

**Affiliations:** a Dentist, Department of General Dentistry, Affiliated Haikou Hospital of Xiangya Medical College, Central South University, Hainan Provincial Stomatology Center, Hainan, China. Performed periodontal treatment, performed data analysis.; b Dentist, Hainan TaiKang Bybo Dental Hospital LTD, Hainan, China. Performed data analysis.; c Dentist, Department of General Dentistry, Affiliated Haikou Hospital of Xiangya Medical College, Central South University, Hainan Provincial Stomatology Center, Hainan, China. Performed data analysis.; d Dentist, Department of General Dentistry, Affiliated Haikou Hospital of Xiangya Medical College, Central South University, Hainan Provincial Stomatology Center, Hainan, China. Performed periodontal treatment, wrote the manuscript.

**Keywords:** Er:YAG laser, inflammation, laser therapy, periodontitis, photobiomodulation

## Abstract

**Purpose:**

The effectiveness of using different laser therapy strategies in adjunct to scaling and root planing (SRP) for treatment of periodontitis remains unclear. This study compared the treatment outcome of SRP and its combination with Er:YAG laser and/or photobiomodulation on patients with severe periodontitis.

**Materials and Methods:**

A total of 50 patients were included, whose teeth were divided into 4 quadrants: 1. SRP+Er:YAG laser irradiation+photobiomodulation treatment; 2. SRP+Er:YAG laser irradiation treatment; 3. SRP+photobiomodulation treatment; 4. SRP treatment only. An Er:YAG laser at powers of 50 or 40 mJ and an Nd:YAG laser at 50 mJ were used. Patients were followed-up for 3 months. Periodontal clinical parameters (probing depth [PD], clinical attachment level [CAL], plaque index [PLI] and bleeding index [BI]), inflammation factors (melatonin and MMP-8 levels) and pain intensity were compared among the 4 quadrants.

**Results:**

The quadrant treated by SRP combined with Er:YAG laser irradiation and photobiomodulation had statistically significantly lower PD (p = 0.0081 at 1 month; p < 0.0001 at 3 months), CAL (p = 0.003), PLI (p = 0.0011) and BI levels (p = 0.005 at 1 month; p = 0.0236 at 3 months) compared with the other 3 quadrants. In addition, melatonin (p = 0.0006 at 1 month; p = 0.0002 at 3 months) and MMP-8 levels (p = 0.0012; only at 1 month) were also statistically significantly higher.

**Conclusion:**

Of the treatments tested here, SRP combined with Er:YAG laser irradiation and photobiomodulation had the best short-term outcome for severe periodontitis.

Periodontitis is an infectious disease caused by pathogenic dental biofilm, which can lead to many detrimental consequences, including loss of periodontal connective tissue, resorption of alveolar bone and formation of periodontal pockets. It is a disruption of the innate defense surveillance and tissue maintenance of the subgingival plaque biofilm^6,7,12,13^ and is the main cause of tooth loss in adults. At present, the successful treatment of periodontitis relies on the complete removal of periodontal pathogenic microorganisms and their toxic products from the root surface and periodontal soft tissue, as well as the neutralization of host pro-inflammatory cytokines.^11^ Scaling and root planing (SRP) is a traditional treatment strategy for periodontitis. However, this method is limited by the incomplete removal of root surface tartar and infected tissues due to root surface depressions and root bifurcation lesions. In addition, smear layer and scratches are easily formed on the root surface after conventional curettage and root surface leveling, which affects the reattachment of periodontal tissue. Moreover, periodontal pathogens can penetrate and persist in the periodontal pockets to escape host immune responses and conventional antibacterial drugs. Re-colonisation of bacteria in the periodontal tissue after treatment increases the recurrence rate of the disease.

Recently, various types of lasers have been applied in periodontal treatment, among which Nd:YAG laser and Er:YAG laser are the two most commonly used ones. The laser beam acts on the target tissue to produce different biological effects, which are mainly determined by the specific emission wavelength of the laser and the optical characteristics of the target tissue.

Studies have shown several positive disinfection effects of laser treatment, including effective removal of the inflammed tissue, preservation of the non-inflammatory periodontal pocket epithelium, and effective removal of root surface tartar, plaque and diseased cementum.^32^ However, due to the different experimental design and Nd:YAG laser parameters used, other studies found inconsistent results.^27^ Although Nd:YAG laser combined with SRP therapy is not necessarily more effective than SRP alone in treating periodontitis, it can be used as an auxiliary means to improve the composition of microorganisms in subgingival plaque.^27^ In addition, Nd:YAG laser-assisted SRP can reduce the depth of periodontal pockets, but compared with SRP alone, there is no statistically significant difference in the improvement of clinical attachment loss between the two groups.^15^

Er:YAG laser is a solid-state laser with a wavelength of 2940 nm. Its wavelength is in line with the peak laser absorption rate of water and hydroxyapatite, and can cut tooth tissue of various stiffness and minimise the thermal effect to adjacent tissues. Therefore, Er:YAG laser can effectively remove the infected tissues in the periodontal pocket and provide bactericidal and antibacterial effects, generating minimal thermal side effects without smear layer formation.^25,26^ Moreover, the root surface after Er:YAG laser treatment is more conducive to the attachment of periodontal ligament cells compared with traditional treatment.^17^

At present, Er:YAG laser is mainly used in periodontal treatment to assist SRP in clinical practice. Due to differences in experimental design and laser parameters, the outcome comparison between Er:YAG laser-assisted traditional treatment and traditional debridement remains ambiguous. Rotundo et al’s split-mouth clinical trial^22^ showed that the combination of Er:YAG laser (150 m J/pulse; 10 Hz) and SRP do not further improve the clinical treatment outcome compared with SRP alone. On the other hand, Yilmaz et al^33,34^ showed that Er:YAG laser (10 Hz, 30 mJ/pulse and 20 Hz, 50 mJ/pulse) combined with SRP can greatly improve pocket probing depth (PD) and clinical attachment level (CAL) 3 months after treatment.

Photobiomodulation refers to a low-power laser that does not cause irreversible damage to the target tissue when directly irradiated. The stimulating effect of photobiomodulation on biological tissues has the following characteristics: different doses can generate either a stimulating or inhibitory effect; the stimulus has a cumulative effect, and the sum of multiple small doses of radiation is comparable to the biological effect caused by a large dose of radiation; the stimulus follows a parabolic pattern, where the response decreases sharply after reaching a plateau. Photobiomodulation therapy can promote several cellular events, including local periodontal blood circulation, chemotaxis and migration, proliferation and differentiation of periodontal ligament cells, so as to promote wound healing.^21^ Aykol et al^3^ used photobiomodulation to assist periodontal non-surgical treatment and found that the combined treatment can provide greater improvement in the clinical periodontal indicators at 1, 3, and 6 months after surgery.

In periodontal treatment, high-energy laser treatment can remove pathogenic bacteria and diseased tissues in the periodontal pocket, while photobiomodulation can use its biological stimulation function to inhibit periodontal inflammation and promote wound healing. Therefore, the present study intends to broaden the application of lasers in periodontal treatment by exploring the treatment outcome of different combinations of laser treatment with different intensities.

## Materials and Methods

### Patients

All 50 included patients suffered from grade C periodontitis, at stages II or III, based on based on the new classification scheme for periodontal diseases.^4^ The inclusion criteria were (1) at least two sites in each quadrant of the full mouth with PD ≥ 5 mm and CAL ≥ 5 mm; (2) at least 50% of the teeth with alveolar bone resorption ≥30% and bleeding or periodontal pus; (3) at least 20 remaining teeth, with at least one molar in each quadrant; (4) no periodontal treatment within the past 6 months. Patients were excluded if they (1) had taken antibiotics, non-hormonal anti-inflammatory drugs and immunosuppressants within the past 3 months; (2) had systemic diseases or were pregnant; (3) were smokers; (4) did not agreed to participate in the study. All patients received oral hygiene and ultrasound supragingival cleaning 1 week before the SRP and laser treatment.

### Study Design

The study was approved by the ethics committee of Haikou Hospital (SC20200144) and was performed in accordance with the Helsinki declaration. All included patients signed the written consent form. The study followed a single-blinded, randomized design and was performed by an operator who was blinded to the treatment methods. All teeth were divided into 4 quadrants, each of which corresponded to 1 treatment group. Group 1 received SRP+Er:YAG laser irradiation+photobiomodulation treatment. Group 2 received SRP+Er:YAG laser irradiation treatment. Group 3 received SRP+photobiomodulation treatment. Group 4 received SRP treatment only. All patients were treated by the same experienced periodontist. Clinical parameter examination and gingival crevicular fluid collection were performed by another experienced periodontist who did not know the grouping information before treatment and 1 and 3 months after treatment. No check-up or cleaning was performed other than at these time points.

### Examination

The measured periodontal clinical parameters included plaque index (PLI), PD, CAL and bleeding index (BI). The Florida electronic probe system was used for the examination. PLI was assessed based on previous criteria.^28^ Specifically, 0 = no plaque at the gingival margin; 1 = thin plaque at the gingival margin, but can only be seen by scraping the tooth surface with a probe; 2 = medium plaque at the gingival margin or adjacent surface; 3 = large plaque deposits in the gingival sulcus or gingival margin area and adjacent surfaces. Bleeding was induced by gently inserting a periodontal probe into the periodontal pocket. BI was assessed 30 s after the probe was removed and scored as follows: 0 = healthy gums, no inflammation or bleeding; 1 = changes in the color of the gums caused by inflammation, no bleeding after probing; 2 = slight bleeding after probing; 3 = bleeding followed a linear pattern along the gingival margin after probing; 4 = bleeding overflowed the gingival sulcus; 5 = bleeding without probing. Gingival crevicular fluid (GCF) was collected prior to, and 1 and 3 months after treatment. Levels of melatonin and matrix metalloproteinase-8 (MMP-8) were measured using a commercially available ELISA kit (Thermo Fisher; Waltham, MA, USA).

All patients received oral hygiene instructions and ultrasound supragingival cleaning prior to treatment. Ultrasound subgingival curettage was performed with an ultrasonic scaler (P5, SATELEC; Paris, France). Root surfaces were smoothed with manual curettage instruments (Gracey periodontal curettes 5/6, 7/8, 11/12, and 13/14; Hu-Friedy; Chicago, IL, USA).

An Er:YAG laser (M002-6A/2, Fotona; Ljubljana, Slovenia) was applied with water irrigation for 30 s to remove calculus from the root surface. The Er:YAG laser was delivered into the periodontal pockets using a tip with the following parameters: 0.4 mm diameter, 14 mm length, micro short pulse (MSP). When removing granulation tissue, the Er:YAG laser was applied at 40 mJ and 20 Hz on the inner wall of the periodontal pocket, with otherwise the same tip settings. The Nd:YAG laser (50 mJ, 10 Hz, 0.6 mm diameter, 320 µm fiber tip, HSM-III, Sichuan Aerospace Sid Control & Guide; Chengdu, China) was used to irradiate the inner wall of the periodontal pockets for 30 s. Laser protocols were chosen based on our experience and a previously published study.^35^ Pain intensity was recorded 1 and 7 days after treatment using the visual analogue scale (VAS).^10^ Cefuroxime axetil was prescribed for some patients for pain relief. No mouthwash was prescribed.

### Statistics

Statistical analysis was performed with GraphPad 8.0 (San Diego, CA, USA). Normal distribution of data was verified with the Kolmogorov-Smirnov normality test. One-way ANOVA and Student’s t-test were used to compare the parameters among different treatment groups. Statistical significance was defined as p < 0.05.

## Results

**Fig 1:**
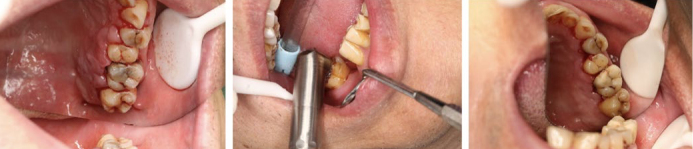
Example images of the affected teeth before (A), during (B) and after (C) the laser therapy.

A total of 50 patients were included in the present study. Figure 1 illustrates affected teeth before, during and after laser therapy. Basic demographic characteristics are given in Table 1. Patients’ age was between 40 and 60 years. Sex distribution was rather even. More included patients suffered from stage III than stage II periodontitis.

**Table 1:** Basic demographic characteristics

Characteristics	n = 50
Age (mean ± SD)	47.6 ± 7.8
Gender (male:female)	26:24
Periodontitis stage (n % of total)	
Stage II	18 (36%)
Stage III	32 (64%)

In terms of periodontal parameters, PLI, PD, CAL and BI were all similar among the 4 quadrants before treatment (Table 2). PLI was statistically significantly lower in treatment quadrants involving SRP combined with laser (quadrant 1, 2 and 3) 3 months after treatment compared to SRP-only treatment (quadrant 4) (p = 0.0011; Table 2). In contrast, the PLI after treatment with SRP combined with Er:YAG and photobiomodulation (quadrant 1) was comparable to that of SRP combined with either laser alone (quadrant 2 and 3) 3 months after treatment (p = 0.707) (Table 2). PD was statistically significantly lower upon addition of laser treatment (quadrants 1, 2 and 3) at both 1 (p = 0.0081) and 3 months (p < 0.0001) after treatment (Table 3). Importantly, the combination of Er:YAG and photobiomodulation (quadrant 1) further reduced PD compared with laser treatment only (quadrants 2 and 3) (p < 0.0001) (Table 2). The same outcome was also observed for CAL at 3 months after treatment time (p = 0.003) (Table 2). For BI, quadrant 1 was statistically significantly better than the other 3 quadrants 1 month after treatment (p = 0.005), while the difference became marginal at the 3-month time point (p = 0.0236) (Table 2).

**Table 2:** Periodontal clinical parameters

	Quadrant 1 (SRP+Er:YAG+ photobiomodulation)	Quadrant 2 (SRP+Er:YAG)	Quadrant 3 (SRP+ photobiomodulation)	Quadrant 4 (SRP)	p-value (all groups)	p-value (Q1, Q2 and Q3)
PLI (mean ± SD)						
Pre-treatment	2.0 ± 0.8	2.0 ± 0.8	2.1 ± 0.8	2.0 ± 0.8	0.9425	0.9611
1 month	2 ± 0.8	2.2 ± 0.8	2 ± 0.8	2.1 ± 0.8	0.721	0.5444
3 months	0.9 ± 0.8	1.0 ± 0.8	1.0 ± 0.9	1.6 ± 1.2	0.0011	0.707
PD (mm, mean ± SD)						
Pre-treatment	7.2 ± 1.3	7 ± 1.4	6.8 ± 1.4	6.9 ± 1.4	0.5996	0.4699
1 month	6.1 ± 1.4	6.8 ± 1.7	6.3 ± 1.6	7.1 ± 1.5	0.0081	0.0997
3 months	3.6 ± 1.2	5.1 ± 1.5	5.0 ± 1.6	5.7 ± 1.7	<0.0001	<0.0001
CAL (mm, mean ± SD)						
Pre-treatment	6.9 ± 1.4	7.2 ± 1.5	6.9 ± 1.2	7.3 ± 1.3	0.2929	0.5068
1 month	6 ± 1.4	6.5 ± 1.6	6.5 ± 1.9	6.8 ± 1.7	0.0953	0.2514
3 months	4.1 ± 1.4	5.0 ± 1.4	4.7 ± 1.4	5.8 ± 1.7	<0.0001	0.003
BI (mean ± SD)						
Pre-treatment	3.3 ± 1.2	3.7 ± 1.2	3.5 ± 1.2	3.3 ± 1.1	0.2529	0.2757
1 month	3.0 ± 0.8	3.5 ± 1.1	3.7 ± 1.2	3.7 ± 1.2	0.0095	0.005
3 months	2.1 ± 1.4	2.5 ± 1.1	2.4 ± 1.2	2.9 ± 1.5	0.0236	0.1897

**Table 3:** Melatonin and MMP-8 levels

	Quadrant 1 (SRP+Er:YAG+ photobiomodulation)	Quadrant 2 (SRP+Er:YAG)	Quadrant 3 (SRP+photobiomodulation)	Quadrant 4 (SRP)	p-value (all groups)	p-value (Q1, Q2 and Q3)
Melatonin (pg/ml, mean ± SD)						
Pre-treatment	10.0 ± 1.1	9.7 ± 1.3	10.0 ± 1.2	9.8 ± 1.2	0.4045	0.2792
1 month	11.4 ± 1.4	11.0 ± 1.2	10.9 ± 1.2	10.4 ± 0.8	0.0006	0.1906
3 months	14.0 ± 1.1	13.1 ± 1.6	13.3 ± 1.9	12.6 ± 1.5	0.0002	0.0165
MMP (ng/ml, mean ± SD)						
Pre-treatment	49.7 ± 8.4	49.8 ± 9.8	51.3 ± 8.5	48.1 ± 8.2	0.3612	0.6127
1 month	49.7 ± 7.9	44.6 ± 8.0	44.3 ± 8.6	47.0 ± 7.8	0.0025	0.0012
3 months	39.4 ± 6.6	41.3 ± 5.9	41.0 ± 5.6	42.0 ± 7.2	0.2136	0.2562

Melatonin and MMP-8 levels in the GCF were comparable among the 4 quadrants before treatment (Table 3). The melatonin level statistically significantly increased in laser treatment groups (quadrant 1, 2 and 3) 1 (p = 0.0006) and 3 months (p = 0.0002) after treatment (Table 3). In addition, the combination of Er:YAG and photobiomodulation (quadrant 1) further increased the melatonin level 3 months after treatment (p = 0.0165) (Table 3). For the MMP-8 level, a statistically significant difference was only observed at the 1-month time point (p = 0.0012) (Table 3).

In terms of pain intensity, no statistically significant difference was observed among the 4 quadrants 1 day after treatment (p = 0.8008 and p = 0.6343, respectively) as assessed by VAS (Table 4). Seven days after treatment, laser treatment quadrants (1, 2 and 3) were statistically significantly less painful than the SRP-only quadrant (p = 0.0355) (Table 4).

**Table 4:** Pain intensity

	Quadrant 1 (SRP+Er:YAG+ photobiomodulation)	Quadrant 2 (SRP+Er:YAG)	Quadrant 3 (SRP+ photobiomodulation)	Quadrant 4 (SRP)	p-value (all groups)	p-value (Q1, Q2 and Q3)
VAS (mean ± SD)						
1 day	3.9 ± 1.9	4.0 ± 2.0	4.3 ± 2.0	4.1 ± 2.0	0.8008	0.6343
7 days	2.8 ± 1.5	2.9 ± 1.5	2.7 ± 1.4	3.5 ± 1.4	0.0355	0.8269

## Discussion

In the past decade, there have been many studies attempting to define the dental-laser treatment outcome for periodontitis. In addition to ablation of the damaged soft tissue wall of the periodontal pocket, laser therapy can also stimulate healing in the surrounding gingival and bone tissues. However, the results are controversial in terms of whether laser therapy can provide an additional benefit when combined with conventional SRP. A meta-analysis summarizing 12 randomised controlled trials showed that adjunctive laser therapy reduced PD 3 months after treatment.^5^ Similarly, another randomly controlled clinical trial comparing the effectiveness of combined Er:YAG and Nd:YAG laser therapy to SRP suggested that the former may have a particularly beneficial effect in areas that are hard to access, such as deep pockets.^23^ In addition, evidence is available indicating that the combination of Er:YAG and Nd:YAG lasers provide better microbiological and clinical outcomes of moderate to severe periodontitis.^9^ On the other hand, studies comparing Er:YAG laser and sonic debridement in the treatment of periodontitis revealed similar clinical and microbiological outcomes.^16,20^ A randomised split-mouth clinical trial performed on an Italian population concluded a lack of additional benefit of Er:YAG laser with conventional SRP.^22^ Similarly, a 2-year follow-up split-mouth study on a German population obtained consistent CAL gains after treatment with an Er:YAG laser or SRP.^25^ Moreover, a randomised, prospective clinical study on a New Zealand population even revealed better short-term improvement in clinical parameters and patient satisfaction for SRP-treated periodontitis compared with Er:YAG laser debridement.^29^

Here, we compared the treatment outcome of SRP and its combination with Er:YAG laser and/or photobiomodulation. Our results are in line with the studies that favour Er:YAG and Nd:YAG laser therapy as a beneficial adjunct treatment method in addition to SRP for periodontitis. Generally, photobiomodulation has the following parameters: output power from 0.001 to 0.1 W, wavelength from 300 to 10,600 nm, pulse frequency from 0 to 5000 Hz and light intensity from 0.01 to 10 W/cm^2^. The Nd:YAG laser protocol used in the present study (50 mJ, 10 Hz, 1.5 W, 320 µm fiber tip) was within these parameter ranges and therefore can be considered as a type of the photobiomodulation therapy. In the present study, the Nd:YAG laser was chosen over the diode laser due to its reported better efficacy in reduction of dentin hypersensitivity.^31^ We showed that the combined treatment can statistically significantly reduce the amount of dental plaque (reduced PLI), improve the patients’ ability to maintain optimal plaque control (reduced PD), prevent the loss of periodontal tissue support in periodontitis (reduced CAL) and decrease gingival inflammation (reduced BI and increased melatonin and MMP-8 levels). Study heterogeneity, including differences in study design, patient populations and laser powers might be the main reason for these divergent results.

Melatonin is a hormone mainly secreted by the pineal gland, which can passively diffuse into saliva through the blood stream.^14^ It has been shown to be an important enhancer of the immune system by activating monocytes and neutrophils.^19^ In diseased periodontal tissues, especially periodontitis, the melatonin level in GCF drops significantly.^1^ MMP-8 is one of the major proteolytic enzymes released by neutrophils during the inflammatory process. Elevated MMP-8 levels in GCF have been shown to be associated with periodontal health.^24,30^ We show that SRP combined with Er:YAG and photobiomodulation can better adjust the GCF melatonin and MMP-8 levels towards the normal condition compared to SRP alone or combined with one of the lasers, suggesting that the two laser treatments might have a better boosting effect on the immune system.

Several possible mechanisms could underlie the potential beneficial outcome observed in laser-treated quadrants. First, Er:YAG laser can effectively remove granulation tissue to promote bone formation without affecting the osseous tissue.^18^ Second, Er:YAG laser can effectively remove bacterial endotoxins from root surfaces.^8^ Third, laser treatment has been shown to promote and support periodontal ligament fibroblast attachment to root surfaces.^2^

The present study has certain limitations. First, the follow-up period was relatively short. Therefore, long-term effects of the tested treatment strategies are not reported in the present study. Future studies with longer follow-up periods are needed to confirm our findings. Second, all patients were from a single institute, which limited the population diversity. Third, we did not collect data on microorganisms. Fourth, the number of included patients was rather small. However, to the best of knowledge, our study is the first that compared the treatment outcomes of SRP and its combination with Er:YAG laser and/or photobiomodulation on a Chinese population, which will provide valuable support in the treatment of periodontitis.

## Conclusion

In summary, our study suggests that SRP combined with Er:YAG laser and photobiomodulation generally leads to better treatment outcomes in terms of periodontal parameters, inflammation factors and pain intensity for patients with severe periodontitis.
